# *Rustitermes boteroi*, a new genus and species of soldierless termites (Blattodea, Isoptera, Apicotermitinae) from South America

**DOI:** 10.3897/zookeys.922.47347

**Published:** 2020-03-25

**Authors:** Daniel Castro, Joice P. Constantini, Rudolf H. Scheffrahn, Tiago F. Carrijo, Eliana M. Cancello

**Affiliations:** 1 Instituto Amazónico de Investigaciones Científicas SINCHI, Avenida Vásquez Cobo Calles 15 y 16, Leticia, Amazonas, Colombia Instituto Amazónico de Investigaciones Científicas SINCHI Leticia Colombia; 2 Facultad de Ciencias Agrarias, Universidad Nacional de Colombia, Carrera 30 # 45-03, Bogotá D.C., Colombia Universidad Nacional de Colombia Bogotá Colombia; 3 Museu de Zoologia da Universidade de São Paulo, Cx. Postal 42.391, 04218–970, São Paulo, SP, Brazil Museu de Zoologia da Universidade de São Paulo São Paulo Brazil; 4 Fort Lauderdale Research and Education Center, Institute for Food and Agricultural Sciences, University of Florida, 3205 College Avenue, Davie, Florida 33314, USA University of Florida Davie United States of America; 5 Centro de Ciências Naturais e Humanas, Universidade Federal do ABC, Rua Arcturus 03, Jardim Antares, 09606-070, São Bernardo do Campo, SP, Brazil Universidade Federal do ABC São Bernardo do Campo Brazil

**Keywords:** Neotropics, enteric valve, soil-feeder, barcode sequence

## Abstract

We present the description of a new genus and species of soldierless termites from South America. *Rustitermes boteroi* Constantini, Castro & Scheffrahn, **gen. et sp. nov.** can be identified by the morphology of the enteric valve, with six slightly asymmetric cushions, each one forming a central pouch made of scales smaller than those between the cushions. The new genus features two characteristic rows of thick bristles on the interior margin of the fore tibia, and is supported by COI molecular sequence data. This species is distributed from Tobago to northern Argentina.

## Introduction

Soil-feeding termites represent a large part of the termite fauna in Neotropical ecosystems (Ackerman et al. 2009; Bourguignon et al. 2011; Palin et al. 2011; Cancello et al. 2014). Species in the subfamily Apicotermitinae are almost exclusively soil feeders (Bourguignon et al. 2016a), and preferential consumption of different soil components has been suggested as a driver of great diversity in these termites (Bourguignon et al. 2009).

The Apicotermitinae subfamily was first proposed by Grassé and Noirot (1954) and defined by Sands (1972) in the most extensive taxonomic work on this group of termites carried out in Africa. Both morphological and molecular data corroborate the monophyly of Apicotermitinae (Inward et al. 2007; Bourguignon et al. 2017).

The taxonomic work of Sands (1972) described 51 new species, redescribed 9 species, and established 16 new genera. Sands (1972) considered the genus *Anoplotermes* Müller to be exclusively Neotropical. At the time, this was the sole apicotermitine genus of the region.

Taxonomic study of the worker caste has been underwhelming in the Neotropical Region (Rocha et al. 2019), and because all New World Apicotermitinae are soldierless, this subfamily has been historically understudied. Before 2009, only five genera had been described (Fontes 1992; Bourguignon et al. 2010). In recent years however, the development of Apicotermitinae taxonomy in the Neotropical Region has been increased by the description of ten new genera, with enteric valve morphology being of essential diagnostic character for the establishment of new taxa (Scheffrahn 2013; Bourguignon et al. 2016b; Scheffrahn et al. 2017; Castro et al. 2018), although other characters may also be very useful, especially in taxa with less robust enteric valve armature (Acioli and Constantino 2015; Constantini et al. 2018). The diversity of soldierless termites can be high, reaching up to 31 morphospecies for a single primary tropical rainforest (Bourguignon et al. 2013).

Herein we describe *Rustitermes boteroi* gen. nov. et sp. nov. based on the morphology of the imago, worker caste and molecular COI data.

## Material and methods

The material examined in this study is deposited at Museu de Zoologia da Universidade de São Paulo, São Paulo, Brazil (**MZUSP**); Colección de artrópodos terrestres de la Amazonía Colombiana of the SINCHI Institute in Leticia, Amazonas, Colombia (**CATAC**); and at the University of Florida Termite Collection at Fort Lauderdale Research and Education Center, Davie, Florida, United States (**UF**). All the specimens are preserved in 80–85% ethanol; colonies marked with (*) in the material examined contain alates.

Some type material of old species was consulted for comparisons and remarks with *R. boteroi* sp. nov., to avoid generating new synonyms for described species. The abbreviations of the cited institutions are: **AMNH** – American Museum of Natural History, New York, USA; **USNM** – Smithsonian National Museum of Natural History, Washington, D.C., USA; **CMNH** – Chicago Museum of Natural History, Chicago, USA. The species reviewed were: *Anoplotermes bolivianus* Snyder (alate, USNM), *Anoplotermes brucei* Snyder (alate and worker, AMNH), *Anoplotermes distans* Snyder (worker, AMNH), *Anoplotermes gracilis* Snyder (alate, AMNH), *Anoplotermes hondurensis* Snyder (alate, AMNH), *Anoplotermes meridianus* Emerson (worker, AMNH), *Anoplotermes punctatus* Snyder (worker, AMNH), *Anoplotermes pyriformis* Snyder (alate, AMNH), *Anoplotermes rotundus* Snyder (alate and worker, AMNH and USNM), *Anoplotermes subterraneus* Emerson (alates and workers, AMNH and USNM), *Anoplotermes tenebrosus* (Hagen) (alate, AMNH), *Aparatermes abbreviatus* (Silvestri) (alate and worker, AMNH), *Aparatermes cingulatus* (Burmeister), and *Aparatermes silvestrii* (Emerson) (workers, CMNH).

The terminology used to describe worker mandibles follows Sands (1972) and Deligne (1999), with some modification, while worker digestive tube descriptions follow Noirot (2001).

In Sands (1972, fig. 2), a variation of the tooth that he calls the “marginal subsidiary” is represented, which may or may not be hidden by the molar prominence (depending on the position it occupies), and suggests that the development of this tooth is a useful generic characteristic. The same tooth is called *premolar* by Deligne (1999) and *molar* by Krishna (1968). Traditionally, the term “subsidiary” is used to designate the structure present at the base of the apical tooth in the left or right mandible in some non-Termitidae families, which could generate some misunderstanding in the literature. In addition, a process not reported in the literature, closer to M3, was observed in the left mandible of some Apicotermitinae alates (Sands 1972). Therefore, we propose to call this a “pre-molar process” for the structure closest to M3 and a “molar process” for the structure closest to the molar prominence, stressing that both processes are part of the molar region (see Fig. 2C, MP).

The mandibles were examined on a microscope slide in PVA medium, after adding a cover glass and pressing them gently into position, as shown in Figure 2C, D. Nevertheless, we examined the mandibles in every possible position before separating them from the head, in order to undertake a careful examination of the “pre-molar process” and “molar process”.

The terms used for pilosity are comparative: bristles are stiff hairs with well-marked bases; spine-like bristles are shorter and thicker than bristles; hairs are shorter and thinner than bristles and without conspicuous bases.

Workers and imagos were examined in a petri dish filled with 80% ethanol, whereas the dissection of the enteric valve (EV) was done with two no. 20 minuten pins (BioQuip, Rancho Dominguez, CA).

The EV was detached from the paunch (P3) and all the food particles were removed by gentle manipulation. The extracted EV was inserted in a drop of PVA mounting medium (BioQuip, Rancho Dominguez, Cat. #6371A) and then gently massaged with the side of a minuten pin for a few minutes until the EV became detached from the muscles. Afterwards, the EV was transferred on to a microscope slide where, after adding another drop of the same mounting medium, the fully cleaned tubular EV was splayed open before final mounting.

The following morphometric characters were measured as defined by Roonwal (1970) and indicated in parentheses: *for alates*–maximum diameter of compound eyes (48); inter-eye distance (52); maximum diameter of ocellus (55); minimum diameter of ocellus (56); eye-ocellus distance (57); length of pronotum (65); width of pronotum (68); minimum length of forewing without scale (75); maximum length of forewing scale (76); *for alates and workers*–length of head to lateral base of mandibles (5); width of head (17); lengths of pro- and metatibia (85); width of protibia (86); protibia index (53, p.61).

Microphotographs were taken as multi-layer montages using a Leica M205C stereomicroscope for the worker head, fore tibia and mandibles; for the worker EV a Leica CTR 5500 compound microscope was used, controlled by the Leica Application Suite version 3 software.

The distribution map was created using ArcGIS desktop ver. 10.4.1 (ESRI, Redlands, CA). The list of examined material is sorted by country (uppercase), state or province, and locality. Collection data are organized as follows: latitude, longitude, collection date, altitude, collector name, collection, and collection number.

The COI barcoding region (Cytochrome c Oxidase subunit 1) was sequenced for four colonies of *R. boteroi* sp. nov. from Peru, Ecuador, French Guiana and Paraguay. DNA extraction and PCR were performed by the Canadian Centre for DNA Barcoding (BOLD systems), following standard high-throughput protocols (deWaard et al. 2008). The PCR employed the primers LepF1 and LepR1 (Hebert et al. 2003), which generated 622 to 652bp. To infer the relationship of *Rustitermes* gen. nov. with the other Neotropical Apicotermitinae, a Bayesian phylogeny was performed with the COI region. In addition to the four sequences of *Rustitermes boteroi* sp. nov. from colonies, UF.FG411 (BOLD:AAW5963), UF.PA534 (BOLD:ACB7291), UF.EC400 (BOLD:ABA4343) and UF.PU602 (BOLD:ACO6749), 49 GenBank and BOLD sequences were used in the analysis: 35 sequences of Neotropical Apicotermitinae (22 species, 14 genera); eight non-Neotropical Apicotermitinae genera, five non-ApicotermitinaeTermitidae, and one Rhinotermitidae [*Heterotermes crinitus* (Emerson)] as outgroup. The tree was constructed under the same parameters as other recently published papers on Neotropical Apicotermitinae (Carrijo et al. 2015; Castro et al. 2018). Sequence alignment was performed under the MUSCLE algorithm; the substitution model used was the GTR+I+G, selected under the Akaike Information Criterion (AIC) by jModelTest2 (Darriba et al. 2012); the phylogeny was reconstructed with BEAST 1.8.0 (Drummond et al. 2012) under a Yule speciation process. Four 20,000,000 generations Markov chain Monte Carlo (MCMC) searches were conducted independently and combined. Sampling was conducted every 2000 generations. Convergence and stationarity were assessed with Tracer 1.5 (Rambaut et al. 2014) and the first 1000 trees of each run were discarded as burn-in.

## Taxonomy

### Rustitermes

Taxon classificationAnimaliaBlattodeaTermitidae

Constantini, Castro & Scheffrahn gen. nov.

A54E1CA4-9EE3-5A01-BA17-8AC2C2747EDE

http://zoobank.org/A6BB62D4-9A1E-4FAD-A0B3-B16B56D4CB87

#### Type species.

*Rustitermes boteroi* sp. nov.

#### Diagnosis.

Enteric valve with six slightly asymmetrical cushions. Each cushion forming a central pouch made of scales smaller than those between the cushions. Each cushion composed of about 60–80 scales, wider at the base and narrower at the apex. Posterior portion of pads truncated, with 35 to 50 rectangular scales arranged from the middle to the apex of the cushion and increasing in density in this same direction.

**Figure 1.**
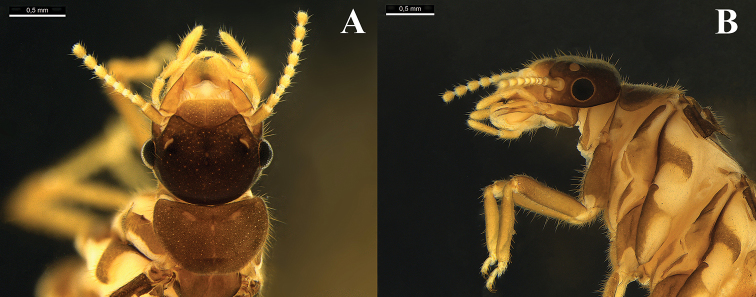
Female imago head capsule, pronotum and fore leg of *Rustitermes boteroi* sp. nov. **A** dorsal view **B** lateral view. Specimen from lot MZUSP 26677. Scale bars: 0.5 mm.

**Figure 2.**
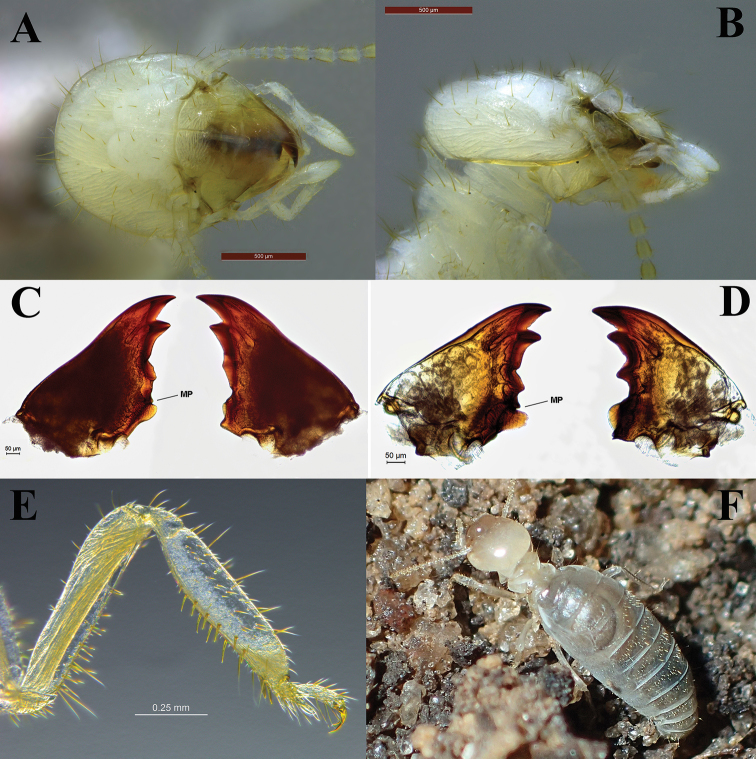
*Rustitermes boteroi* sp. nov. **A, B** worker head capsule in dorsal and lateral view **C** imago mandibles **D** worker mandibles **E** worker right fore tibia **F** live habitus of worker. MP = molar process. Specimens from lot CATAC 1797 (**A, B**), MZUSP 26677 (**C, D**), BO437 (**E**).

***Imago*** (Fig. 1; Table 1). Fontanelle inconspicuous in both sexes, the region of the fontanelle depressed; medium spot slightly conspicuous. Left mandible with apical tooth a little bit larger than M1 + 2; M3 triangular with lateral margins forming an obtuse angle; non-conspicuous premolar process; molar process not hidden by molar prominence (Fig. 2C).

**Table 1.** Measurements (mm) of imagos of *Rustitermes boteroi* sp. nov. from colony MZUSP 26677.

	Female (n = 4)		Male (n = 5)	
	Range	Mean	Range	Mean
Length of head	0.78–0.92	0.83	0.65–0.78	0.75
Width of head with eyes	1.13–1.20	1.18	1.12–1.15	1.12
Maximum diameter of compound eye	0.27	0.27	0.27–0-28	0.27
Inter-eye distance	0.87–0.95	0.93	0.87–0.88	0.87
Maximum diameter of ocellus	0.12	0.12	0.11–0.12	0.11
Minimum diameter of ocellus	0.08–0.09	0.09	0.08–0.09	0.09
Eye-ocellus distance	0.08–0.11	0.1	0.08–0.1	0.09
Length of pronotum	0.57–0.63	0.6	0.55–0.58	0.56
Width of pronotum	1.00–1.08	1.04	0.97–0.98	0.97
Length of forewing with scale	11.60–12.13	11.82	10.40–10.53	10.47
Width of fore tibia	0.13	0.13	0.13	0.13
Length of fore tibia	0.88–0.95	0.93	0.83–0.88	0.86

Head capsule with short, sparse bristles; coloration of head capsule dark brown; frontal marks slightly lighter than rest of head capsule, with poorly defined margins. Pronotum subhexagonal, with anterior margin straight, without central incision; lateral margins very straight and well-marked; pronotum with few sparse bristles and short hairs. Tergites and sternites with short hairs covering the plates. Fore coxa with a set of 4–5 prominent long bristles; inner face of fore tibia with two rows of 6–7 thick bristles.

**Figure 3.**
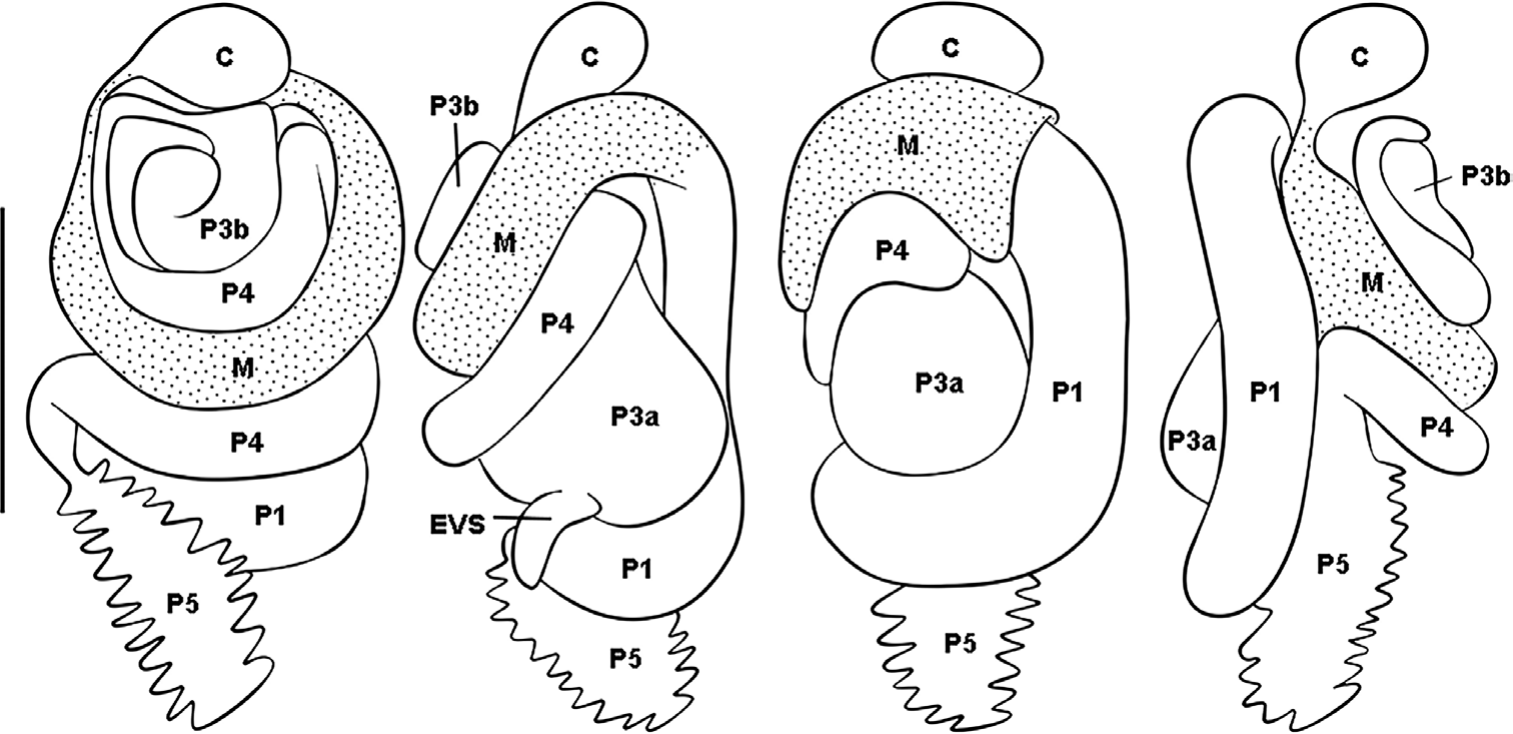
Digestive tube from left to right: dorsal, right, ventral and left views (gut regions indicated: *C* = crop, *M* = mesenteron, *P1* = ileum, *P3a* and *b* = paunch, *P4* = colon, *P5* = rectum, EVS = enteric valve seating). Scale bar: 0.5 mm.

**Figure 4.**
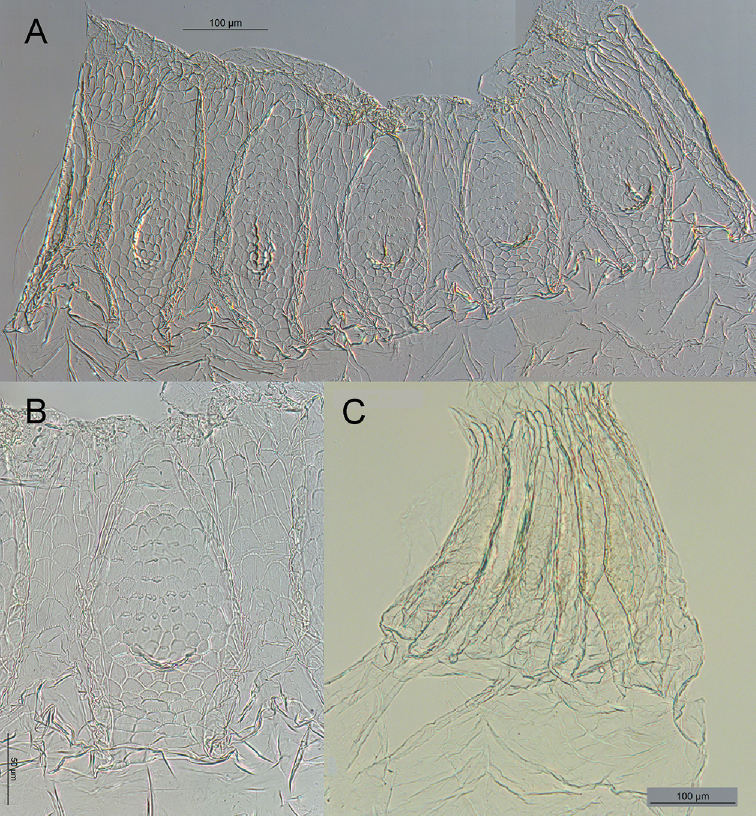
Worker enteric valve of *Rustitermes boteroi* sp. nov. **A** EV fully stretched laterally, showing the six cushions (end cushion bisected) **B** EV detail of smallest cushion in A **C** whole mount EV lateral profile of cushions. Food flow in each image from bottom to top.

***Worker*** (Figs 2–4). Monomorphic. Small and rounded fontanelle; postclypeus rather slightly inflated; head capsule covered with medium and long bristles. Left mandible with prominent apical tooth compared to M1 + 2, triangular M3 with lateral margins forming a right/acute angle, molar process not concealed by molar prominence. Pronotum with long bristles, concentrated along margin of anterior and posterior lobes. Tergites and sternites with dense cover of long bristles, facing the posterior region or upwards. Fore coxa with a set of 4–5 thick bristles; inner face of fore femur with long bristles. Fore tibia moderately inflated, inner face of fore tibia with two rows of 6–7 thick bristles.

Mixed segment (MS) separated from ileum (P1) by a simple transverse junction; P1 of uniform width along entire length, forming an inverted C in ventral view. Enteric valve without armature, with six pyriform cushions of slightly different dimensions, the two largest and two smallest cushions adjacent to each other. The center of each cushion is formed into a lumen-facing pouch consisting of about 60 fringed scales. The remainder of the cushions consists of 50–75 (depending on size of cushion) larger fringed polygons. The cuticle between the cushions is composed of even larger fringed cuboidal scales. All cushions are wider at base (near P1) and narrower at apex (near P3). Tubular and short EV seating. Worker measurements highly variable among and within different colonies (Table 2).

**Table 2.** Measurements (mm) of 9 colonies (n = 10) of *Rustitermes boteroi* sp. nov. L = length, W = width.

	Length of head with postclypeus	Max. width of head	Hind tibia L	Fore tibia W	Fore tibia L	Ratio fore tibia W/L
Holotype	0.95	0.95	0.88	0.18	0.58	0.30
PU602	0.79–0.89 (0.86)	0.96–1.00 (0.99)	0.95–1.04 (0.99)	0.14–0.16 (0.15)	0.74–0.79 (0.78)	0.18–0.21 (0.20)
AG360	0.8–1.05 (0.86)	0.85–1.18 (0.92)	0.53–0.75 (0.63)	0.10–0.15 (0.13)	0.43–0.53 (0.46)	0.24–0.29 (0.28)
BO431	0.82–0.96 (0.90)	1.02–1.09 (1.04)	0.89–0.98 (0.95)	0.14–0.18 (0.16)	0.72–0.77 (0.75)	0.18–0.23 (0.21)
EC400	0.92–0.95 (0.89)	1.0–1.04 (1.01)	0.98–1.04 (1.01)	0.16–0.19 (0.18)	0.77–0.82 (0.80)	0.20–0.24 (0.23)
MZUSP 13712	0.80–0.84 (0.83)	0.98–1.12 (1.04)	0.77–0.88 (0.84)	0.13–0.18 (0.15)	0.63–0.72 (0.67)	0.19–0.26 (0.22)
FG411	0.80–0.85 (0.82)	0.87–0.93 (0.89)	0.68–0.80 (0.74)	0.17–0.20 (0.18)	0.55–0.58 (0.57)	0.33–0,35 (0.33)
PA8	0.82–0.89 (0.85)	0.88–0.95 (0.91)	0.88–0.96 (0.91)	0.16–0.19 (0.18)	0.68–0.75 (0.73)	0.21–0.27 (0.24)
TT1614	0.77–0.88 (0.82)	0.86–0.91 (0.89)	0.88–0.96 (0.90)	0.14–0.18 (0.16)	0.70–0.74 (0.72)	0.19–0.24 (0.22)
CATAC-0954	0.85–1.01 (0.91)	0.93–1.02 (0.99)	0.77–0.85 (0.82)	0.16–0.20 (0.18)	0.65–0.71 (0.69)	0.20–0.24 (0.22)

#### Comparison and remarks.

The digestive tube coiling of the new genus is similar to *Hydrecotermes*, but *R. boteroi* sp. nov. can be differentiated by the worker, which has thick bristles along the inner margin of the fore tibia, absent in *Hydrecotermes*. In the workers, the enteric valve and the digestive tube may be similar to *Aparatermes*, but the cuticle between the cushions in *Aparatermes* does not have cuboidal scales. In *Aparatermes* the insertion of P1 in P3 occurs in dorsal view with a trilobate EV setting, in *Rustitermes* the enteric valve seating (EVS) is not trilobate. Also, the enteric valve of *Aparatermes* has small spines or pointy scales, which are absent in *R. boteroi* sp. nov.; in addition, the EV in *Aparatermes* has the posterior portion of the pads without scales. The imago presents a visible molar process and fore tibia with two rows of thick bristles.

#### Molecular analysis.

**Figure 5.**
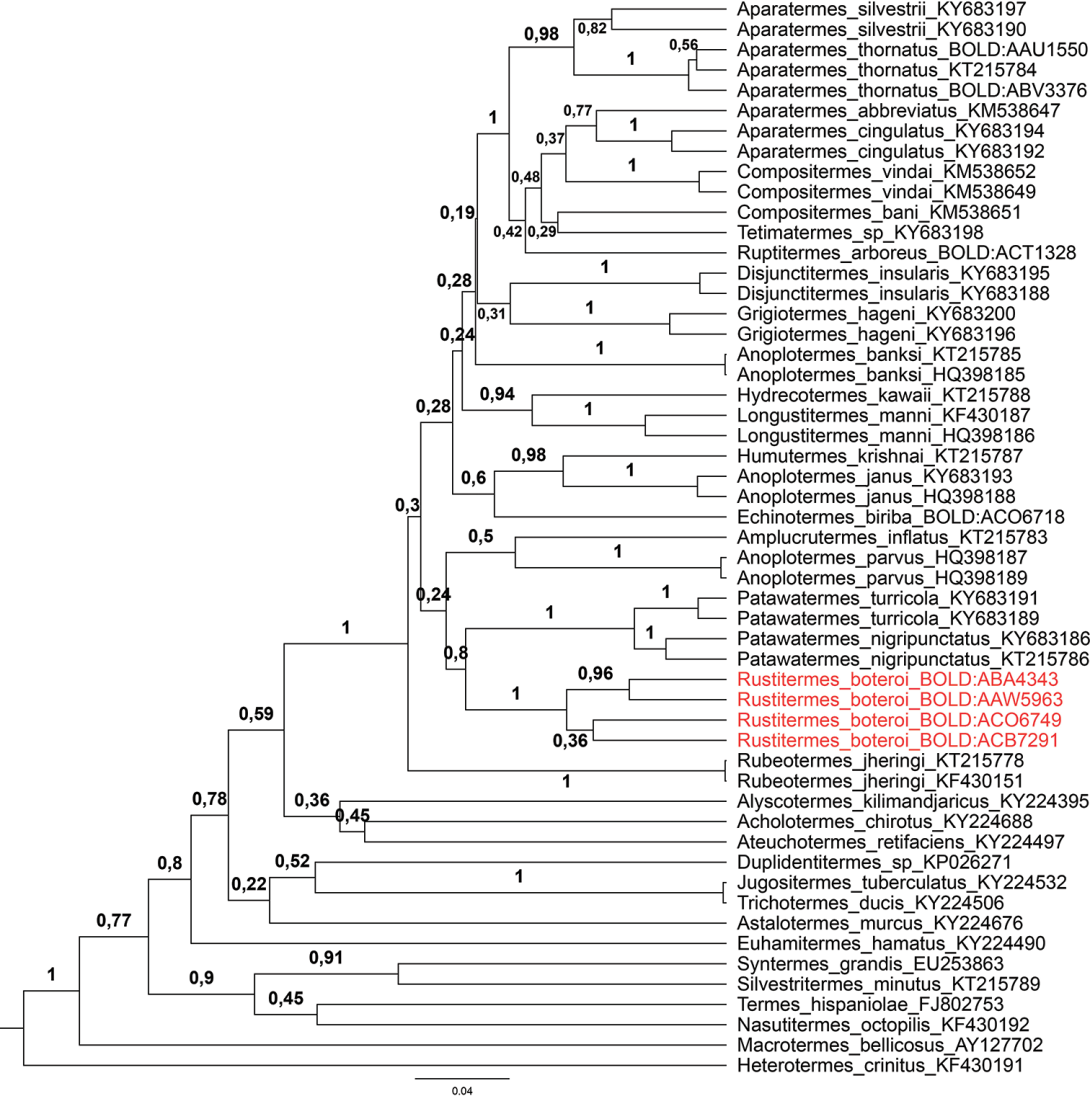
Bayesian phylogenetic tree of the Apicotermitinae subfamily using the COI region. In red, *Rustitermes boteroi* gen. et sp. nov. Branch support is posterior probability.

The Bayesian phylogeny using the COI marker clearly separates *Rustitermes* gen. nov. from the other soldierless termites (Figure 5). The new genus was recovered as sister group of *Patawatermes*, but without high posterior probability support.

#### Etymology.

Named in honor of Michael K. Rust, retired professor of urban entomology at the University of California, Riverside (UCR), and mentor of RHS. Mike encouraged RHS to publish his first taxonomic paper (Scheffrahn and Rust 1983).

### Rustitermes boteroi

Taxon classificationAnimaliaBlattodeaTermitidae

Constantini, Castro & Scheffrahn sp. nov.

4D8E52D7-339E-539F-8286-6FD6B9652BE3

http://zoobank.org/95C95B44-17A3-464D-AC58-ADE9F3BC168D

#### Material examined.

***Holotype*.** Worker from colony labeled as UF no. PU602; the holotype is kept in a separate small vial in the same vial as the paratypes.

#### Type locality.

PERU. *Ucayali*, Nueva Requena, -8.37007, -74.84366.

#### Type repository.

University of Florida, Fort Lauderdale Research and Education Center, Termite Collection in Davie, Florida.

***Paratypes*.** Argentina. *Corrientes*, Santo Tome, (-28.57900, -56.0840), 1.JUL.1998, 93 m, J. Křeček coll. (UF no. AG360). BOLIVIA. *Cochabamba*, Chapare, Villa Tunari, (-18.15343, -60.03293), 26.MAY.2013, 408 m, Chase, Křeček, Mullins, Nishimura, Mangold, and Scheffrahn coll. (UF no. BO85). *Beni*, San Javier, (-14.70207, -64.89097), 29.MAY.2013, 152m, Chase, Křeček, Mullins, Nishimura, Mangold, and Scheffrahn coll. (UF no. BO375); (-14.54909, -64.88964), 29.MAY.2013, Chase, Křeček, Mullins, Nishimura, Mangold, and Scheffrahn coll. (UF no. BO431, BO437). *Santa Cruz*, Roboré, (-18.15343, -60.03293), 31.MAY.2013, 408 m, Chase, Křeček, Mullins, Nishimura, Mangold, and Scheffrahn coll. (UF no. BO738). BRASIL. *Alagoas*, Quebrangulo, (-9.2288, -36.4259), 19.JUN.2000, 780 m, MP Silva coll. (MZUSP 13712). *Bahia*, Conde, (-11.7718, -37.7301), 15.JUN.2016, 78 m, JP Constantini coll. (MZUSP 26648). *Espírito Santo*, Pedro Canário, (-18.3557, -39.8445), 20.JUN.2016, 43 m, JP Constantini coll. (MZUSP 26652); 21.JUN.2016, (MZUSP 26676(a), 26677*). *Paraíba*, João Pessoa, (-7.1480, -34.8614), 01-20.JUN.2000, 66 m, A Vasconcellos coll. (MZUSP 13710, 13711). *Pernambuco*, Recife, Horto Dois Irmãos, (-7.9999, -34.9473), s/d, 88m, A Vasconcellos coll. (MZUSP 13702). COLOMBIA. *Amazonas*, La Chorrera, Lago grande (-2.07066, -72.170611), 28.JUN.2016, 133 m, D. Castro coll. (CATAC-1712); Leticia, (-4.046666, -70.00566), 13.JUL.2018, 126 m, D. Castro coll. (CATAC-3137). *Caquetá*, Belén de los Andaquíes, (+1.3515, -75.81178), 23.APR.2018, 280 m, H Artunduaga coll. (CATAC-3688); (+1.26663, -75.78983), 24.FEB.2016, 252 m, Y. Virguez coll. (CATAC-1793); (+1.63063, -75.90591), 28.JAN.2017, 758 m, D. Castro coll. (CATAC-0954); Florencia, (+1.716694, -75.61369), 29.MAR.2016, 527 m, Y. Virguez coll. (CATAC-1781); San Vicente del Caguan, (+2.03560, -74.91294), 14.APR.2018, 339 m, CP Peña coll. (CATAC-1797). ECUADOR. *Orellana*, Tuptini, (-0.67177, -76.39793), 28.APR.2011, 223 m, Scheffrahn, Chase, Mangold, Křeček, Myles, Nishimura and Setter coll. (UF no. EC400). FRENCH GUIANA. *Cayenne*, Sinnamary, (+5.06314, -52.98479), 13.FEB.2008, 102 m, J. Křeček coll. (UF no. FG411). PARAGUAY. *Central*, Ypacaraí, (-25.38044, -57.20014), 27MAY2012, 248 m, Scheffrahn, Chase, Mangold, Křeček and Myles coll. (UF no. PA8). PERU. *Ucayali*, Nueva Requena, (-8.37007, -74.84366), 29.APR.2014, 185 m, Carrijo, Chase, Constantino, Mangold, Mullins, Křeček, Kuswanto, Nishimura, and Scheffrahn coll. (UF no. PU602, PU613). TRINIDAD AND TOBAGO. *Anse Fourmi*, Manson Hall, (+11.28467, -60.60133), 31.MAY.1996, 472 m, Chase, Mangold, Křeček, and Scheffrahn coll. (UF no. TT619). *Guayaguayare*, Río Claro-Mayaro (+10.23516, – 61,13266), 20.MAY.2003, 41 m, Chase, Mangold, Křeček, and Scheffrahn coll. (UF no. TT1614). VENEZUELA. *Bolívar*, Cantarrana, (+4.46750, -61.59694), 29.APR.2004, 874 m, J. Perozo coll. (UF no. VZ1443.1).

#### Diagnosis.

Unarmed enteric valve with six slightly asymmetrical cushions, each one forms a central pouch made of about sixty scales, smaller than those between the cushions.

***Imago*.** As described for the genus.

***Worker*** (Figs 2–4; Table 2). Monomorphic, head capsule with long and short bristles, with more abundance of long bristles. Head capsule color varying between whitish and yellowish. Antennae with 14 articles densely covered with short hairs and some long bristles. Pronotum with long bristles, concentrated along the margins of the anterior and posterior lobes, with some sparse short bristles in the center of the pronotum. Inner face of fore tibia with two rows of 6–7 thick bristles. Inner face of fore femur with thick bristles. Mesotibia and metatibia with 25–35 long, thick bristles.

Enteric valve without armature, with six pyriform cushions of slightly different dimensions, each cushion consisting of 50–75 (depending on size of cushion) larger fringed polygons. The cuticle between the cushions is composed of even larger fringed cuboidal scales assembled close to P3.

#### Remarks.

See remarks for genus.

#### Ecology and distribution.

**Figure 6.**
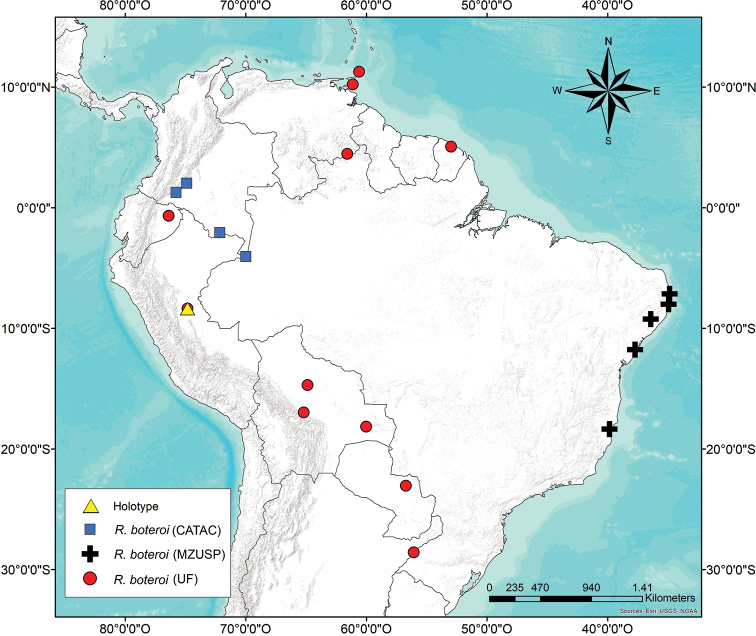
Distribution map of *Rustitermes boteroi* sp. nov. Collection data from the following collections: CATAC (SINCHI Institute), MZUSP (Museu de Zoologia da Universidade de São Paulo) and UF (University of Florida).

This species was collected mainly in soil, although it can also be found at the base of trees or occasionally under pieces of wood or fallen tree limbs above ground. Very common in pastures and open areas; found in young rubber crops in great abundance, less abundant in natural forests. Range: from Trinidad and Tobago to northern Argentina and the Atlantic Forest in Brazil (see discussion below); no known records for Chile and Uruguay (Figure 6).

#### Etymology.

Named in honor of the great Colombian artist Fernando Botero.

## Discussion

Despite its wide distribution and abundance in disturbed sites and open areas *R. boteroi* sp. nov. had not been previously described, indicating the lack of taxonomic work on soldierless termites. Currently, the use of the worker caste for the taxonomic identification of termites has been shown to be increasingly necessary, regardless of the presence of imago or soldier castes (Rocha et al. 2019).

Many other Apicotermitinae species present wide distributions in South America, such as *Compositermes vindai* Scheffrahn, which has been reported from Panama to Paraguay (Scheffrahn 2013), *Aparatermes silvestrii* (Emerson), reported from Trinidad and Tobago to Paraguay (Pinzón et al. 2019), *Longustitermes manni* (Snyder), reported from Honduras to Brazil (Bourguignon et al. 2010), *Tonsuritermes tucki* Constantini and Cancello, reported from Colombia and French Guiana to southern Brazil and Paraguay (Constantini et al. 2018); and, with older records, species such as *Anoplotermes meridianus* Emerson, 1925 and *Anoplotermes parvus* Snyder, 1923, recorded from Central America to Argentina (Bourguignon et al. 2010; Krishna et al. 2013; Constantino 2019). Possibly, many others common species are not yet described, and many others, already described, have unknown ranges. Species with disjunct distributions based on few records probably have much larger distributions, as is the case with *Disjunctitermes* species (Scheffrahn et al. 2017). The New World Apicotermitinae are a typical example of both Linnean and Wallacean shortfalls (Bini et al. 2006).

According to the data presented, *R. boteroi* sp. nov. is widely distributed in the Guiana shield, the Amazon and the Atlantic forest. An effort is needed to identify Cerrado, Caatinga and Amazonian samples (there is abundant material deposited in MZUSP) to determine if this species is present in these areas.

Molecular phylogeny using the COI marker was useful to complement evidence of the separation of *R. boteroi* sp. nov. from the other Apicotermitinae genera. However, this marker alone is not enough to provide a resolved phylogeny allowing to understand the evolution of this group. The new world Apicotermitinae were determined to be monophyletic, but the relationship between most genera had very low branch support, making it impossible to provide deeper discussions.

## Supplementary Material

XML Treatment for Rustitermes

XML Treatment for Rustitermes boteroi
